# Comparative Study of Key Aroma Components in Rice of Different Aroma Types Using Flavor Metabolomics

**DOI:** 10.3390/foods15020200

**Published:** 2026-01-07

**Authors:** Shengmin Qi, Haibin Ren, Haiqing Yang, Lianhui Zhang, Min Zhang

**Affiliations:** 1 School of Food and Health, Beijing Technology and Business University, Beijing 100048, China; 13720035676@163.com; 2 Beijing Engineering and Technology Research Center of Food Additives, Beijing 100048, China; 3 Beijing Advanced Innovation Center for Food Nutrition and Human Health, Beijing 100048, China; 4 Grain Research and Development Center, Nutrition & Health Research Institute, COFCO Corporation, Beijing 102209, China; renhaibin@cofco.com (H.R.); yanghaiqing1@cofco.com (H.Y.); zhanglianhui@cofco.com (L.Z.)

**Keywords:** rice aroma types, GC-IMS, GC-MS, aroma compounds

## Abstract

This study aimed to analyze the volatile organic compounds (VOCs) for different rice aroma types using sensory evaluation, headspace solid-phase microextraction gas chromatography mass spectrometry (HS-SPME-GC-MS), and gas chromatography-ion mobility spectrometry (GC-IMS) techniques, and to explore the material basis for the flavor differences. Based on the sensory evaluation results, rice aroma was categorized into three types, distinguished by their unique aroma compounds. Type A was characterized by a prominent sweet, popcorn aroma, type B by a more prominent cereal and starchy flavor, and type C by a more complex aroma. Untargeted metabolomics analysis using HS-SPME-GC-MS identified and characterized 74 volatile compounds. A comparison of A versus B versus C revealed 8 key aroma compounds, primarily alkanes, aldehydes, ketones, alcohols, and heterocyclic compounds. (E)-2-Octenal, 6-Undecanone, 2-Acetyl-1h-pyrrole, and P-menthan-1-ol in type A gave it a better sweet aroma, Dodecane, 2,6,10-trimethyl-, 1-Octen-3-one, and 2-Methyldecane in type B gave it a better starchy and cereal flavor. 2-Acetyl-1h-pyrrole, Heptacosane, and 1-Propanol in type C contributed to a complex aroma. GC-IMS analysis showed that the fingerprints of rice with different aroma types were significantly different. The VOCs of aroma type A contained (+)-limonene, 2-methylpyrazine, 2-pentanone, ethyl butanoate, n-pentanal, styrene, 1-butanol, 3-methyl-, acetate, 1-hexanal, 1-pentanol, and 2-heptanone, which gave it a better sweet aroma. The VOCs of aroma type C contained 1-octen-3-ol, 2,6-dimethyl pyrazine, 2-acetylpyridine, and ethyl hexanoate, which gave it a better complex aroma.

## 1. Introduction

Rice, one of the major food crops in the world today, serves as the primary food source for more than half the world’s population, particularly in Asia and Africa [1,2]. Rice, domesticated by the Chinese between 13,500 and 8200 years ago [3,4], is now a staple crop worldwide [5]. The United Nations Food and Agriculture Organization reports that rice production is expected to reach a record high of 555.5 million tons (converted into refined rice) in the 2025/26 fiscal year [6]. Changes in the consumer market and advancements in rice breeding have made the flavor of rice a critical indicator of its market price and consumer acceptance. Therefore, the study of rice flavor is particularly important.

Current rice flavor research mainly focuses on two key areas: analyzing volatile compounds in rice and characterizing the aroma quality [7]. The quantitative descriptive analysis (QDA) method is a subjective evaluation technique mainly used to establish descriptive words, which are then used to analyze the aroma attributes of the fragrant rice [8,9,10]. Gas chromatography, supplemented by related pretreatment and detection techniques, is currently the main objective flavor evaluation method [11]. Numerous studies have confirmed that the flavor of rice is jointly determined by the concentrations, thresholds, and interactions of different types of volatile compounds. Currently, more than 500 volatile components have been detected and identified in rice [12,13], mainly including hydrocarbons, alcohols, aldehydes, esters, ketones, phenols, heterocyclic compounds, and so forth [14,15]. Multiple studies using sensory evaluation and flavor metabolomics have analyzed the volatile organic compounds (VOCs) in rice/millet [16,17], confirming that only a select few are responsible for the distinct and significant flavor profile of rice [18]. Studies have also reported the effects of different processing conditions on the flavor of rice [14]. These key aroma components undergo concentration changes during breeding, planting, drying, storage, processing, preservation, cooking, and oral chewing processes, and the compounds exhibit different flavors due to varying concentrations, thereby influencing the overall flavor of rice [19].

Currently, rice is classified into two main categories based on aroma: fragrant rice and nonfragrant rice. The key differences in volatile components between fragrant and nonfragrant rice include 2-acetyl-1-pyrrolidine (2-AP), 2-octene, isopentanol, 2-ethyl-1-hexanol, 1-octen-3-ol, and so forth [20]. 2-AP, first identified in 1982 [21], has a strong popcorn aroma [22,23,24]. It is responsible for the characteristic aroma compound of fragrant rice [25]. Several studies have also reported on the aroma types of rice. Lu et al. [12] used gas chromatography–mass spectrometry (GC-MS) to analyze the volatile components in fragrant rice from China and Thailand. They divided fragrant rice into three types (popcorn flavor, corn flavor, and lotus seed flavor) and determined the main aroma-active substances of each type. Dong et al. [26] used GC-MS, Gas chromatography–olfactometry (GC-O), and multivariate analysis to analyze the aroma chemical components of six different types of rice from India, Thailand, and South Korea. They identified the main compounds causing aroma differences. Zhou et al. [19] collected nine samples of rice with different aromas, established an aroma description word bank based on QDA, identified four aroma types, and analyzed the flavor substances in each aroma type. At present, the rice aroma research is mainly focused on fragrant rice. However, fragrant and nonfragrant rice have their own specific aromas for consumers, and the differences in their flavor substances result in certain differences in their aroma types. Thus, the classification of rice with different aroma types due to different flavor substances needs further systematic research.

Taking into account the significant influence of the aroma of raw rice on consumers’ purchasing decisions, this study focuses on raw rice as the research object, aiming to verify the feasibility of rice aroma differentiation based on the main varieties available in China. The material basis of rice aroma differences was systematically analyzed using sensory evaluation, GC-MS, and GC-IMS analytical methods. The aromas of different Chinese rice varieties were differentiated and the key flavor substances were explored, providing a reference for the quantitative evaluation and standard upgrade of rice aroma and offering high-quality products to consumers.

## 2. Materials and Methods

### 2.1. Chemicals and Reagents

2-Methyl-3-heptanone, 4-Hydroxy-2,5-dimethylfuran-3-one, Butyraldehyde, 1-Octanol, 2-Acetyl-1-pyrrolidine were procured from Tokyo Chemical Industry Co., Ltd. (Shanghai, China). A mixed standard of n-alkanes (C9–C23) (chromatographic grade, purity ≥ 99.5%) was bought from Shanghai Anpu Laboratory Technology Co., Ltd. (Shanghai, China). n-Ketones [2-butanone, 2-pentanone, 2-hexanone, 2-heptanone, 2-octanone, and 2-nonanone (all analytical grade)], phenethyl alcohol were procured from Shanghai Aladdin Biochemical Technology Co., Ltd. (Shanghai, China). Starch, Toothpick, pasteurized milk, quaker oatmeal, cream style sweet corn were procured from Wal-Mart Stores (Beijing, China). Ultrapure water was prepared by a Hokee Company (Hefei, China, HK-DI-10/20/30 model).

### 2.2. Sample Collection

11 different types of commercial raw rice, all with distinct aromas, were purchased between July and October 2024. Prior to the experiments, each sample was divided into three portions for subsequent research. These samples were vacuum-packed and stored at 4 °C. All experiments were completed within 2 months of rice purchase. All samples were prepared before each test, kept fresh in the refrigerator, and then brought to room temperature for evaluation. The samples were assigned three random numbers. Table 1 presents the numbers and variety information of the samples.

### 2.3. Sensory Evaluation of Rice Flavor

According to the requirements of GB/T 16291.1-2012 (National Standard, 2012) [27], a rice sensory evaluation panel was formed based on previous findings [28,29,30]. 10 participants regularly engaged in sensory analysis of cereal products, primarily adults over the 20 years of age with at least two years of experience in the sensory evaluation of rice products, were enrolled in the study. Three sensory training sessions were conducted. In the first one, panelists with rich experience in rice descriptive sensory analysis generated aroma descriptors for raw rice samples studied. For the second session, the panelists performed a sensory evaluation of the samples based on their own subjective perceptions and recorded some different aroma descriptors. In the last session, all sensory panelists were asked to participate in a discussion of sensory descriptors screening and deletion. Ultimately, ten aroma attributes (starchy, grainy, floral, Popcorn, sweet, grassy, woody, oatmeal, dairy, corn) were determined to be most effective in characterizing the rice and highlighting key differences among them. After the training period, the quantitative description analysis was performed using the screened sensory descriptors and the provided standard references (Table 2) [19]. The strength of each descriptor ranged from 0 to 9 [31], with 0 representing no sensation and 9 representing a strong sensation (0.5 is the minimum increment).

The sensory experiment was conducted in an air-conditioned laboratory at 25 °C with good ventilation. The rice samples (20 g) were placed in a plastic cup and marked with a three-digit code according to Table 1. The participants were asked to quickly select the descriptors they thought were suitable to describe the flavor attributes of the rice. Samples were presented at intervals of approximately 5 min. The results of each aroma descriptor of samples were averaged for statistical analysis.

### 2.4. Flavor Metabolomic Analysis by HS-SPME-GC-MS

Flavor metabolomic analysis was performed on 11 different types of commercial raw rice. The method used headspace solid-phase microextraction gas chromatography mass spectrometry (HS-SPME-GC-MS), with an Agilent system consisting of a 7890B chromatograph and a 5977B mass spectrometer (Agilent Technologies, Santa Clara, CA, USA). The aroma compounds were extracted using a Supelco solid-phase microextraction fiber [50/30 µm (Divinylbenzene (DVB)/Carboxen (CAR)/Polydimethylsiloxane (PDMS), StableFlex (2 cm)], and the volatile compounds were separated using a DB-Wax capillary column (30 m × 250 μm × 0.25 μm, Agilent Technologies, Santa Clara, CA, USA).

#### 2.4.1. Metabolite Extraction

First, 0.816 μg/μL of 2-methyl-3-heptanone standard solution was prepared using deionized water as the solvent. The rice samples (2000 ± 40 mg) were placed in 20 mL headspace bottles, and 1 μL of 2-methyl-3-heptanone was added as an internal standard. Headspace injection conditions included incubation at 60 °C, preheating for 15 min, adsorbing for 30 min, and desorption for 4 min.

#### 2.4.2. GC-MS Analysis

The volatile substances were analyzed using a DB-Wax chromatographic column and an Agilent 7890B gas chromatography–5977B mass spectrometry system (Agilent Technologies, Santa Clara, CA, USA). The system used a nonsplit injection mode, with high-purity helium as the carrier gas. The pre-injection port purge flow rate was 3 mL/min, and the column flow rate was 1 mL/min. The initial temperature was controlled at 40 °C for 4 min, and then increased at a rate of 5 °C/min to 245 °C and maintained for 5 min. The injection port temperature was 250 °C, the transfer line temperature was 250 °C, the ion source temperature was 230 °C, and the quadrupole temperature was 150 °C. The electron impact ionization energy was 70 eV. The mass spectrometry scan range was set at *m*/*z* 20–400, and the solvent delay time was 2.37 min. After the measurement, the mass spectrum of the volatile compounds were then compared with data from the NIST 2017 database. Then, the actual retention index (RI) value is calculated according to the relevant research methods of the same laboratory [32,33]. The RI were compared with the values reported in the literature. When the difference is <50, it was inferred that the two match. The volatile compounds were quantified (semi-quantitative analysis) by dividing the peak areas of the compounds of interest by the peak area of 2-methyl-3-heptanone as internal standard. Each experiment with the samples was conducted triplicately.

#### 2.4.3. Calculation of Relative Odor Activity Value

The relative odor activity values (ROAV) were performed to evaluate the specific contribution of each compound to the overall aroma, which was well reported in previous studies [34]. ROAV was mainly used to quantitatively evaluate the contribution of each volatile substance to the overall flavor of the test sample and thus identify key aroma-active compounds. A high ROAV is also indicative of the great contribution of a component to the overall flavor of the sample. The compounds with ROAV ≥ 1 were the principal aroma compounds of the sample, whereas those with 0.1 ≤ ROAV < 1 had a important modifying effects on its overall flavor. ROAV was calculated as described previously [35,36].

### 2.5. GC-IMS Analysis

GC-IMS analysis of volatiles was performed using an Agilent 490 GC (Agilent Technologies, CA, USA) and a FavourSpec IMS instrument (Gesellschaft für analytische Sensorsysteme (G.A.S.), Dortmund, Germany) as described previously [37,38]. First, 5000 ± 100 mg of rice sample was ground at low temperature and placed in a 20 mL headspace vial. The headspace injection conditions included incubation at 60 °C for 20 min and splitless injection with an injection volume of 500 µL, an injection speed of 500 rpm, and an injector temperature maintained at 85 °C. A capillary column (15 m × 0.53 mm × 1.0 μm, MXT-5; Restek, Bellefonte, PA, USA) was used, with a column temperature of 60 °C and 99.99% nitrogen as the carrier gas. A programmed pressure was employed with an initial flow rate of 2.0 mL/min, which was maintained for 2 min and then linearly increased to 10.0 mL/min for 8 min, 100.0 mL/min for 10 min, and 150.0 mL/min for 10 min. The chromatographic run time was set at 30 min, and the inlet temperature was maintained at 80 °C. The volatile compounds were identified based on the drift time and RI according to the National Institute of Standards and Technology (NIST) and G.A.S. databases. These measurements were performed in triplicate. The IMS conditions were as follows: ionization source, tritium (3H); drift tube length, 53 mm; electric field strength, 500 V/cm; drift tube temperature, 45 °C; drift gas, high-purity nitrogen (≥99.999%); flow rate, 75 mL/min; and positive ion mode. Each experiment with the samples was conducted in triplicate.

The retention index (RI) was calculated using n-ketones C4–C9 (Sinopharm Chemical Reagent Beijing Co., Ltd., Beijing, China) as external references by the Laboratory Analysis View (LAV) software(LAV version 2.0.0; G.A.S.) in the GC-IMS device. The volatile compounds were tentative identified based on comparison of RI and the drift time with the NIST library and IMS database retrieval software obtained from G.A.S (Dortmund, Germany, version 2.0.0). Finally, the intensities of the volatile compounds were analyzed according to the peak volumes of the selected signal peaks by Gallery Plot analysis (v.1.0.7, G.A.S.) [37]. Visual and quantitative comparisons of volatility differences between samples were performed using the Gallery plot plug-in. Two-dimensional top views and three-dimensional fingerprints were constructed using the Reporter plug-in.

### 2.6. Statistical Analysis

Chroma Time-of-Flight (TOF) 4.3X software of LECO Corporation (St. Joseph, MI, USA) and NIST database were used for raw peak extraction, data baseline filtering and calibration of the baseline, peak alignment, deconvolution analysis, peak identification, integration, and spectrum matching of the peak area [39].

The Reporter, Gallery Plot, and Dynamic Principal component analysis (PCA) plug-ins in LAV software were used to generate two- and three-dimensional spectra, difference spectra, fingerprints, and PCA plots of volatile components for comparing VOCs between samples. In order to avoid deviations between measurements, the drift time of sample spectra was normalized relative to RIP drift time, which proceed automatically in the LAV software.

Excel and Origin 2021 software were used for statistical analysis, including principal component analysis (PCA), cluster analysis, spider plots, and histograms.

## 3. Results

### 3.1. Sensory Evaluation and Aroma Classification of Rice

**Figure 1:**
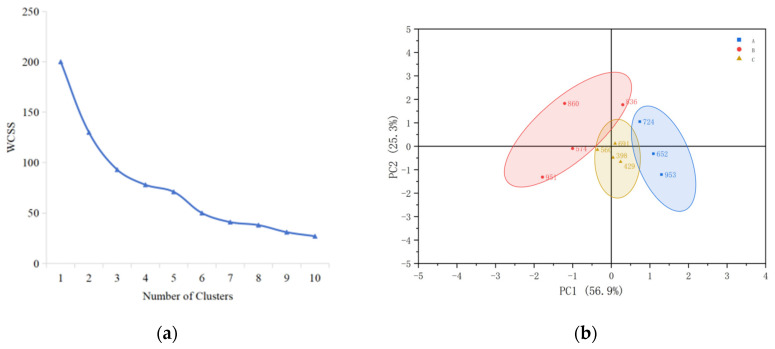
Ward hierarchical cluster analysis of sensory evaluation: (**a**) relationship between the number of clusters and WCSS, (**b**) cluster diagram of sensory evaluation of 11 rice samples, A: aroma type A rice; B: aroma type B rice; C: aroma type C rice.

The flavor sensory properties of 11 different rice types were evaluated, and their aroma profiles were clustered using the Ward hierarchical clustering method. The number of clusters was determined by the elbow method using the Within-Cluster Sum of Squares (WCSS). The results are presented in Figure 1a. As the number of clusters increases, the WCSS value decreases. The decrease is rapid from class 1 to class 3, and after the number of clusters reaches 3, the curve of WSS changes gradually and becomes flat, which is identified as the “elbow” point. To maintain the tightness within the clusters while ensuring the degree of segmentation, cluster 3 is selected as the best clustering number. The 11 rice samples were divided into 3 major groups. The first group comprised three samples, and the second and third groups comprised four samples each. The results are shown in Figure 1b.

**Figure 2:**
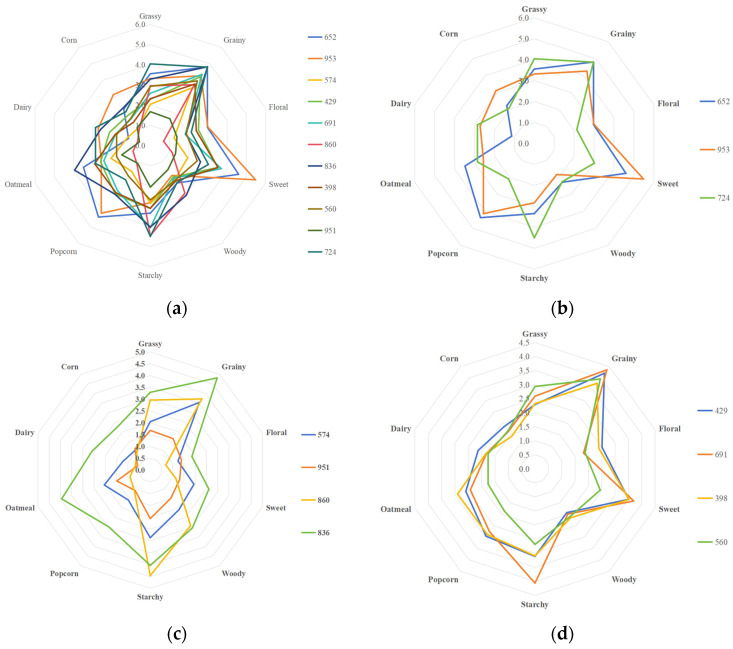
Aroma profile spider plots of rice of different aroma types: (**a**) all rice samples; (**b**) aroma type A rice; (**c**) aroma type B rice; and (**d**) aroma type C rice.

The aroma characteristics of each group of rice were analyzed, and a flavor profile spider plots was drawn (Figure 2), illustrating different aromas. (1) The first group included samples 652, 953, and 724. Their sweet, popcorn, cereal, and grassy aromas were more prominent, and no obvious floral aroma was observed. This group of samples was defined as the sweet aroma type (aroma type A). (2) The second group included samples 574, 951, 860, and 836. The most prominent aromas of this group were cereal and starchy, whereas their sweet and popcorn aromas were significantly reduced compared with other samples. Other sensory attributes were not obvious, so this group of samples was defined as the cereal aroma type (aroma type B). (3) The third group included samples 429, 691, 398, and 560. Their cereal, sweet, and starchy aromas were higher, and the aroma of this group was complex. Therefore, this group of samples was defined as a complex aroma type (aroma type C).

### 3.2. Analysis of Volatile Aroma Compounds in Different Rice Aromas Based on HS-SPME-GC-MS

#### 3.2.1. VOCs in Rice with Different Aroma Types

HS-SPME-GC-MS was used to analyze the volatile components of 11 aromatic rice samples. Table 3 presents the volatile compounds of the three aromatic rice types. HS-SPME-GC-MS analysis of 11 samples identified 74 volatile compounds. The number of volatile compounds varied among rice with different aromas. In addition, 52 volatile compounds with flavor characteristics were identified, which constituted 70.27% of the identified aroma compounds.

**Figure 3:**
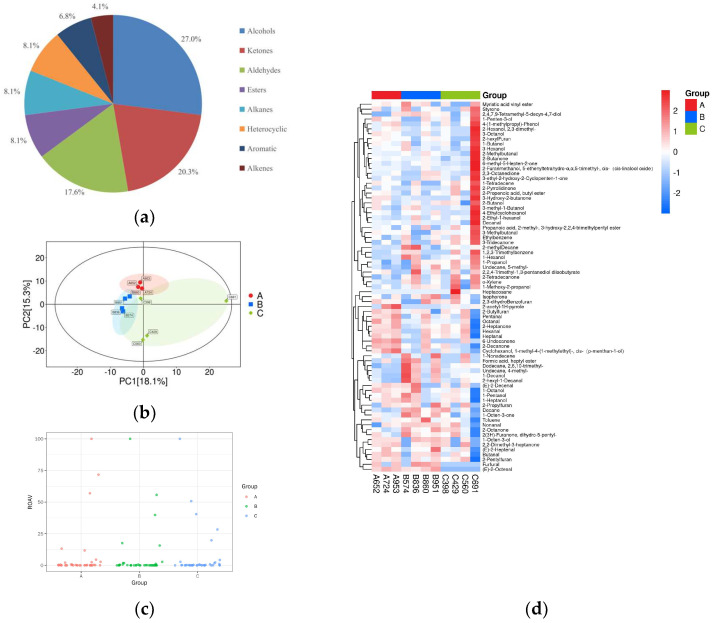
Multivariate statistical analysis of volatile compounds: (**a**) pie chart of metabolite classification proportions; (**b**) cluster analysis: A: aroma type A rice; B: aroma type B rice; C: aroma type C rice; (**c**) relative odor activity values; (**d**) heat map of hierarchical clustering analysis for all groups.

HS-SPME-GC-MS analysis of 11 samples characterized 74 volatile compounds into 8 categories, as shown in Figure 3a. Of these, alcohols accounted for 27%, followed by ketones molecules for 20.2%, aldehydes for 17.5%, esters, alkanes and heterocyclic for 8.1%, and aromatics compounds for 6.7%, and alkenes for 4%.

A PCA of 11 rice varieties was performed based on volatile aroma compounds, as shown in Figure 3b. All samples were within the 95% confidence interval (Hotelling’s T2 ellipse), confirming clear differentiation between the three aroma types. Based on 74 volatile compounds, the samples were sorted into three distinct clusters: the first cluster comprised samples 652, 953, and 724; the second primarily comprised samples 574, 951, 860, and 836; and the third primarily comprised samples 429, 691, 398, and 560. This clustering was consistent with the cluster analysis results from the sensory evaluation. The ROAV of the volatile aroma compounds was calculated to determine which volatile compounds had the greatest impact on the overall flavor of the tested samples. This allowed the identification of key aroma compounds and more effective analysis of their contribution to the flavor of rice varieties, as shown in Figure 3c. An ROAV scatter plot was drawn based on the ROAV to visualize the distribution of the ROAV of flavor substances in different groups. Each point in the figure represents a substance. The horizontal axis of the ROAV scatter plot represents different sample groups, and the vertical axis represents the ROAV of flavor substances in these groups. A cluster analysis was conducted on all the volatile flavor substances of the samples, and a cluster heat map was drawn. Figure 3d shows the cluster heat map of the total content of volatile compounds in rice samples of different aroma types, indicating that volatile aroma compounds differed significantly among these samples.

#### 3.2.2. Analysis of the Differential Volatile Profiling of Three Rice Cultivars

The 8 volatile compound values with the greatest differences were screened out by performing analysis of variance (ANOVA) on the volatile flavor compound data of the three rice types and based on the principle of ANOVA *p*-value < 0.05 (Table 4).

Table 4 shows that the main differential volatile compounds were alkanes, aldehydes, ketones, alcohols, and heterocyclic compounds, and so forth. (E)-2-Octenal and 2-Acetyl-1h-pyrrole exhibited significant aroma expression in rice. Among these, (E)-2-Octenal mainly contributed to the fatty, herbal, fresh, green, fruity, and nutty flavors [19]. 2-Acetyl-1h-pyrrole contributed to a nutty flavor, 1-Octen-3-one contributed to a herbal flavor [19,42,50].

**Figure 4:**
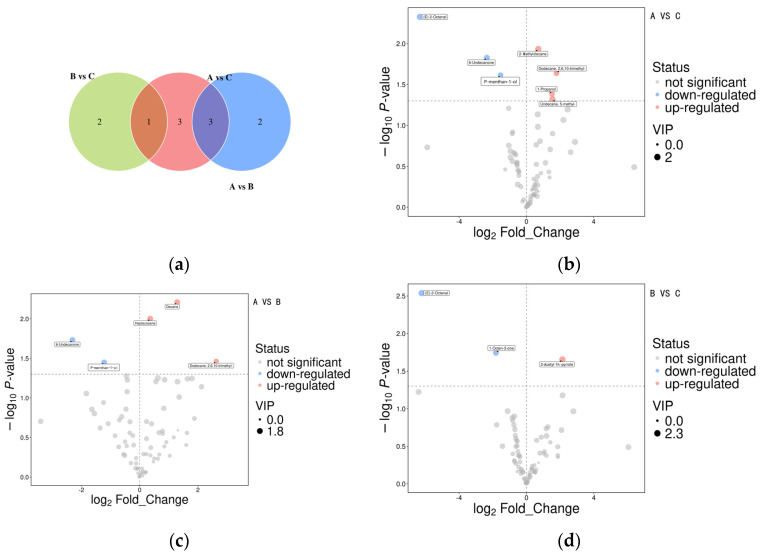
Differential flavor substances among aroma type A, B, C. (**a**) Venn analysis of differential metabolites among different comparison groups, (**b**) Volcano plots of differential metabolites of A versus C, (**c**) Volcano plots of differential metabolites of A versus B, (**d**) Volcano plots of differential metabolites of B versus C.

The volatile flavor data for the three aroma types were analyzed pairwise using Student *t* tests and Orthogonal Partial Least Squares-Discriminant Analysis (OPLS-DA) model analysis of variance. Figure 4 shows the differential volatile compounds across different rice aroma types based on the Student *t* test (*p* < 0.05) and the variable importance in the projection (VIP) of the OPLS-DA model greater than 1. In Figure 4a, different circles represent different comparison groups. The numbers in the overlapping area indicate the number of common differentially expressed metabolites between the comparison groups, and the numbers in the nonoverlapping area indicate the number of unique differentially expressed metabolites. A total of 7 common differentially expressed metabolites between groups A and C, 5 between groups A and B, and 3 between groups B and C. Across the comparison groups, 3 common differentially expressed metabolites between A versus C and A versus B groups, and 1 between A versus C and B versus C groups.

Figure 4b–d shows the results of the differential metabolite screening as volcano plots, with each dot representing a metabolite. The abscissa represents the fold change (logarithm to base 2) of each substance in the comparison group, and the ordinate represents the *p* value (negative logarithm to base 10) of the Student *t* test. The size of the scatter plot represents the VIP value of the OPLS-DA model, with larger scatter plots indicating higher VIP values. The significantly upregulated metabolites in Figure 4b–d are represented in red, the significantly downregulated metabolites are shown in blue, and the metabolites with no significant difference are indicated in gray. Figure 4b–d ashows that the A versus B comparison group had a higher number of differentially expressed metabolites, indicating a greater difference between aroma types A and B. In the three comparison groups, the flavor of the differential volatile substances in aroma type B was relatively weaker and not distinctive, indicating it had a less distinctive flavor than aroma types A and C, and A and C had better sweet, fruity, and other aromas compared with aroma type B.

**Figure 5:**
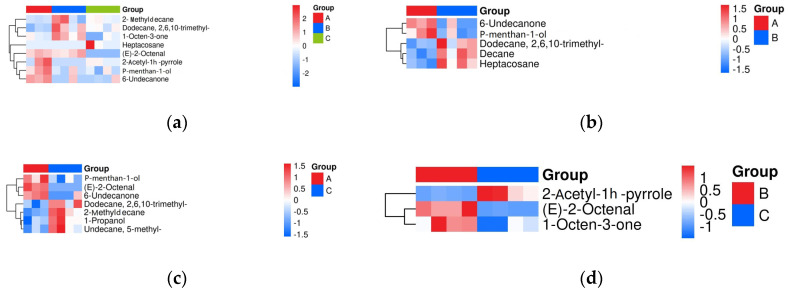
Hierarchical cluster analysis heat map among aroma type A, B, C. (**a**) A versus B versus C comparison group, (**b**) A versus B comparison group, (**c**) A versus C comparison group, and (**d**) B versus C comparison group.

The Euclidean distance was used to quantify the differences in metabolites among different comparison groups. The data were clustered using the complete linkage method, and the results were presented as a heat map, shown in Figure 5.

The different colors in the graph on the horizontal axis represent different rice aroma types, the vertical axis represents the differential metabolites, and the different color blocks represent the relative expression levels of the corresponding metabolites. Red indicates a higher expression level of the substance, and blue indicates a lower expression level. Figure 5a presents the data of the A versus B versus C comparison group. The expression levels of (E)-2-Octenal, 6-Undecanone, 2-Acetyl-1h-pyrrole, and P-menthan-1-ol were high in the aroma type A rice. Among these, (E)-2-Octenal had a nutty, fatty, herbal, fresh, and green aroma [19]. This also better explained the sensory results. Compared with aroma types B and C, aroma type A had a better sweet and grassy aroma. The expression levels of Dodecane, 2,6,10-trimethyl-, 1-Octen-3-one, and 2-Methyldecane were high in aroma type B, among which 1-Octen-3-one had a herbal, mushroom aroma [51]. This also better explained the sensory results that aroma type B had a more prominent starchy and grainy taste compared with aroma types A and C. In aroma type C, the expression levels of Heptacosane were high, and this group had richer differentiated volatile characteristics. This also confirmed that the aroma of this group was complex and was defined as a complex aroma type.

Figure 5b–d represents the data of the A versus B, A versus C, and B versus C comparison groups, respectively. In addition, the expression levels of 6-Undecanone, P-menthan-1-ol in type A were higher than in B in the A versus B comparison group. The expression levels of Dodecane, 2,6,10-trimethyl-, Decane and Heptacosane were higher in aroma type B than in aroma type A. However, the flavor characteristics of these volatile substances were not obvious, indicating that aroma type B had no prominent flavor characteristic. In the A versus C comparison group, the expression levels of 2-Methyldecane, 1-Propanol, and 5-Methylundecane were higher in aroma type C than in aroma type A. Among these, 1-Propanol had a better peanut aroma [51]. Therefore, type C had a better nutty and fruity aroma than types A. In aroma type B versus aroma type C comparison group, the expression levels of (E)-2-Octenal and 1-Octen-3-one were higher in aroma type B. (E)-2-Octenal contributed nutty, fatty, herbal, fresh, and green aroma characteristics [19]. The expression levels of 2-Acetyl-1h-pyrrole were higher in aroma type C. 2-Acetyl-1h-pyrrole had a bready, nutty, and caramel aroma [51]. Therefore, type C had a better nutty flavor than types B.

### 3.3. Analysis of Volatile Odor Components of Rice by GC-IMS Spectroscopy

#### 3.3.1. Volatile Compounds Identified by GC-IMS

**Figure 6:**
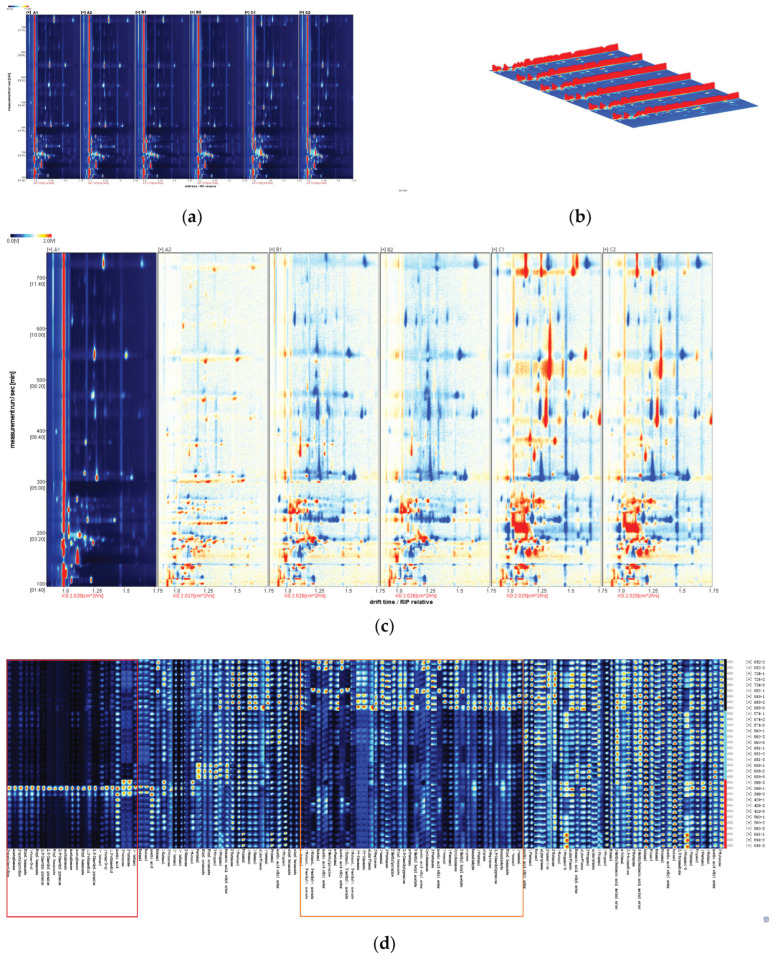
GC-IMS analysis of volatile compounds in 11 samples of the 3 aroma types A, B, and C. Volatile fingerprints (**a**), gallery plots (**b**), and comparison of differences using A1 as a reference (**c**). Fingerprint of volatile components of rice with different flavors (**d**).

The volatile components of the three rice cultivars were determined using GC-IMS, and fingerprint patterns and cluster heat maps were obtained to more intuitively distinguish the differences in volatile components among different types of fragrant rice. As shown in Figure 6, the ion migration time and the position of the reactive ion peak (RIP) were normalized; the ordinate represents the retention time of the gas chromatography; the abscissa represents the ion migration time, and the vertical line at the abscissa 1.0 is the RIP. Figure 6a shows that most of the signals appeared in the retention time of 100–800 s and the drift time of 1.0–1.75. As shown in Figure 6b, the X, Y, and Z axes represent the ion drift time, retention time, and peak intensity of the volatile substances, respectively, which were used for identifying volatile substances [52]. In addition, some volatile compounds such as 1-pentanol, 1-propanol, and acetic acid ethyl ester produced multiple spots or signals, which were interpreted as protonated monomers, trimers, polymers, and proton-bound dimers in the fingerprint area, which could not be observed using GC-MS [53].

Figure 6a,b shows that the fingerprints of different aroma types of rice samples differed significantly after normalization of ion drift time and reactive ion peak position, with the signal intensities for A, B, and C showing considerable differences.

The fingerprints of two samples were randomly selected for each aroma type, using A1 as a reference. This reference sample was compared with the fingerprints of the other aroma types to generate a differential spectrum, as shown in Figure 6c. Higher concentrations are displayed in red, concentrations equal to the reference value are displayed in white, and concentrations below the reference value are displayed in blue. Although VOCs signal intensities within the same aroma type (A1/A2, B1/B2, and C1/C2) were not significantly different, they were significantly different among different aroma types, with A and C showing a significant difference. Samples C1 and C2 displayed more red dots compared with samples A1 and A2, indicating a more intense aroma and flavor profile for aroma type C. Samples B1 and B2 displayed more blue dots, indicating a milder, more balanced flavor profile, consistent with the sensory evaluation and flavor metabolomics results.

The fingerprint identification method was adopted to display the volatile chemical information in the samples for a deeper understanding of the flavor characteristics of different aroma types of rice, as shown in Figure 6d. In this figure, each row represents the signal peak of the volatile components of each sample, and each column represents the signal peak of a specific volatile chemical substance in different samples. The color of the signal peak represents the substance. These graphs present the complete volatile compound profile of each sample in an easily understandable manner, highlighting the differences between samples [54,55]. The volatile chemical fingerprints of aroma types A, B, and C were significantly different. The two distinct regions in the figure represent the differences in the volatile chemical fingerprints between different flavor types. The red region represents primarily alcohols, heterocyclic compounds, and ketones, whereas the orange region represents primarily esters and aromatic hydrocarbons. In the red region, the volatile chemical fingerprint of flavor type C was significantly higher than that of A and B, indicating that C had a higher content of alcohols, heterocyclic compounds, and ketones, giving it a richer flavor profile. In the orange region, the volatile chemical fingerprint of flavor type A was significantly higher than that of B and C, indicating that A had a higher content of esters and aromatic hydrocarbons, giving it a better sweet and fragrant flavor [56]. Flavor type B had fewer volatile aroma compounds than flavor types A and C, which is why the flavor of type B was softer than the other two aroma types.

#### 3.3.2. Differential Analysis of Volatile Compounds Based on GC-IMS

Table 5 lists all the volatile substances in the three fragrant rice types and their peak intensities. A total of 55 volatiles were detected and identified, including 14 ketones, 13 alcohols, 12 esters, 8 aldehydes, 4 heterocyclic compounds, 3 aromatic hydrocarbon compounds, and 1 other compound. The number of these categories differed from the results of GC-MS. A total of 14 VOCs were identified as monomers and dimers. These chemicals had high proton affinity or concentration, generating several signals in the analysis [57]. The aforementioned data showed that the volatile aroma compounds of the three different aromatic rice types had significant characteristics [51]. The samples of aroma type A contained higher contents of (+)-limonene (lemon- and orange-like flavors), 1-hexanol (grassy flavor), 2-methylpyrazine (nutty, coffee, and cocoa-like flavors), 2-pentanone (fruity, floral, and grassy flavors), 3-methyl butyl acetate, cyclohexanone, ethyl butanoate (a unique fresh fruity flavor), n-pentanal (fruity and floral flavors), styrene (floral and sweet flavor), 1-butanol (fruity and caramel flavors), 3-methyl-acetate 1-hexanal (grassy and fruity flavors), 1-pentanol (vanilla and sweet flavors), and 2-heptanon (coconut, sweet, and fruity flavors), indicating a better sweet aroma. In contrast, the samples of aroma type C contained higher contents of 1,2-ethanediol and 1-octen-3-ol (mushroom, lavender, and green-like flavors), 2,6-dimethyl pyrazine (peanut butter and peanut flavors), 2-acetylpyridine (popcorn flavor), acetophenone, and ethyl hexanoate (fruity and green flavors), indicating a better overall aroma. The various volatile flavor substances in aroma type B, were between those in aroma types A and C, without any significant outstanding features, which was consistent with the results of sensory evaluation and flavor metabolomics.

### 3.4. Comparison of Different Volatile Compounds in Rice with Different Aroma Type Based on HS-SPME-GC-MS and GC-IMS

The differential flavor and compound analysis showed that the three rice aromas could be differentiated in sensory evaluation, HS-SPME-GC-MS, and GC-IMS.

From a sensory perspective, the main differential flavors of the three aromas included sweetness, woodiness, popcorn, cereal, and corn flavors. The main feature of aroma type A was the prominent sweetness, and the main feature of aroma type B was the prominent woodiness, cereal, and starch flavors. Aroma type C was more comprehensive than aroma types A and B, with a balanced focus on cereal, sweetness, starch, and popcorn flavors.

From the perspective of differential volatile substances, the main differential compounds included alkanes, aldehydes, ketones, alcohols, and heterocyclic compounds.

Aldehydes are an important type of volatile compounds in rice. These compounds have a low odor threshold and give rice pleasant grassy, citrus, and fresh grassy flavors at low levels. They are an important component of rice flavor. Aldehydes are decomposition products of lipid oxidation [58]. Among these aldehydes, the key volatile aroma compounds of rice are octanal [59], pentanal (valeraldehyde), and heptanal [48], which were also found in the differential compounds of the three aroma types. Studies have shown that heptanal, octanal, and so forth are formed from linoleic acid precursors [60]. In this study, the presence of aldehydes such as octanal and n-pentanal resulted in the prominent sweet flavor of aroma type A compared with aroma type B.

Alcohols are the second major VOCs identified in rice. Methanol, 1-propanol, 1-butanol, 1-pentanol [60], and 1-octen-3-ol are the key volatile aroma compounds of rice. They were all detected in the control sample and were the most abundant besides aldehydes [15]. Alcohols (1-octen-3-ol, hexanol, 1-octanol, etc.) have higher olfactory thresholds, contributing to rice’s mild aromatic profile [61,62]. The floral and fruity aroma of rice is attributed to alcohols [1]. Among all alcohols, 1-octen-3-ol is produced during lipid oxidation. It has a high content, a low threshold, and the smell of wild mushrooms, and contributes the most to the aroma of rice [63]. Previous reports have shown that 1-octen-3-ol can be derived from the peroxide of linoleic acid [64]. In this study, the contents of alcohols such as 1-octen-3-ol and 1-propanol were higher in aroma type C than in aroma type A, which is why aroma type C exhibited a richer flavor than aroma type A.

Ketones are the third largest class of volatile components found in japonica rice. These compounds contribute a pleasant fruity flavor to the rice [61,65]. The main ketones identified in this study were 2-butanone and 2-heptanone, which gave cooked rice a unique fruity and pleasant aroma [66]. 2-Heptanone is produced from oleic acid [60]. In this study, the presence of ketones such as 2-heptanone resulted in a prominent sweet aroma in aroma type A compared with aroma types B and C.

Esters can give a fruity and floral aroma to rice [67]. However, they generally have a relatively high taste threshold, thus contributing little to the flavor of rice [68]. Ethyl acetate, octanoate acetate, and other esters affect the flavor of rice, making it more intense [15]. Therefore, the esters in type A contributed more to its sweet aroma.

Other volatile compounds contributing to odor include heterocyclic compounds such as pyridines and pyrazines. Heterocyclic compounds are mainly formed through nonenzymatic browning reactions or Maillard reactions [69]. Although these compounds have low concentrations, their thresholds are also low, so they contribute significantly to rice flavor, often imparting sweetness, nutty flavors, and beany notes to rice [1,15]. Aroma type C contains more heterocyclic compounds such as pyridines and pyrazines than aroma type A, which is one of the reasons why aroma type C has a more complex aroma.

Because hydrocarbon compounds have a higher threshold, their contribution to the overall aromatic properties of rice is limited [70]. Therefore, although some aroma type contain higher levels of hydrocarbons, their impact on their distinctive flavors is minimal.

## 4. Discussion

This study used sensory evaluation, HS-SPME-GC-MS, GC-IMS, and data analysis to systematically investigate the differences in flavor and volatile aroma compounds among different rice samples. The study also explored the key aroma components differentiating rice aroma types, dissecting the molecular basis for these flavor differences. The main findings were as follows:

1. Feasibility of distinguishing rice aromas based on sensory evaluation: Cluster analysis based on sensory evaluation data categorized rice aromas into three types: sweet aroma type (A), cereal aroma type (B), and complex aroma type (C). Type A exhibited a prominent sweet and popcorn aroma, type B exhibited a more prominent grainy and starchy aroma, and type C exhibited a more complex aroma.
2. Nontargeted metabolomics analysis using HS-SPME-GC-MS: The findings revealed the diversity and differences in volatile components of rice with different aroma types. A total of 74 volatile compounds were identified and classified into three aroma types through PCA, consistent with the sensory results. In the A versus B versus C comparison group, 8 major differential aroma compounds were identified, primarily including alkanes, aldehydes, ketones, alcohols, and heterocyclic compounds. (E)-2-Octenal, 6-Undecanone, 2-Acetyl-1h-pyrrole, and P-menthan-1-ol in type A gave it a better sweet aroma, Dodecane, 2,6,10-trimethyl-, 1-Octen-3-one, and 2-Methyldecane in type B gave it a better starchy and cereal flavor. 2-Acetyl-1h-pyrrole, Heptacosane, and 1-Propanol in type C contributed to a complex aroma.
3. GC-IMS analysis: The results revealed the diversity and differences in volatile components of rice with different aroma types. The fingerprints of rice samples with different aroma types showed significant differences. Compared with aroma type A, aroma type C was stronger and aroma type B was milder and more balanced, consistent with the results of sensory evaluation and flavor metabolomics. Samples of aroma type A contained (+)-limonene, 2-methylpyrazine, 2-pentanone, ethyl butanoate, n-pentanal, styrene, 1-butanol, 3-methyl-, acetate, 1-hexanal, 1-pentanol, and 2-heptanone, contributing to a more sweet aroma. Samples of aroma type C contained 1-octen-3-ol, 2,6-dimethyl pyrazine, 2-acetylpyridine, and ethyl hexanoate, contributing to a more complex aroma. The volatile aroma compounds in aroma type B fell between those in aroma types A and C, with no significant standout characteristics, consistent with the results of sensory evaluation and flavor metabolomics.

This study attempted to validate the feasibility of rice aroma differentiation. A comprehensive analysis was conducted on mainstream commercially available rice varieties to determine the molecular factors behind their distinct aromas, using sensory evaluation, flavor metabolomics. Combining complementary methods such as HS-SPME-GC-MS and GC-IMS, a more complete profile of volatile compounds was obtained, which helped distinguish different rice aromas and revealed their molecular basis. Future investigations should build on this study by increasing sample diversity, integrating metabolomics and machine learning technologies to further analyze rice flavor differences and their formation pathways, thereby guiding processing innovation and quality improvement.

## Figures and Tables

**Table 1:** Information on commercially available rice samples.

No.	652	953	574	429	691	724	860	836	398	560	951
varieties	Nanjing46	Daohuaxiang	Kongyu131	Suijing18	jasmine rice	Longyang16	Suxiu	Taixiangjing1402	Jihong6	Ningxiangjing9	Zhongkefa5

**Table 2:** The rice aroma descriptors and definitions.

Descriptor	Definition	Reference Substance
Starchy	Aroma of bread and flour	Boiled starch:water (1:5) and cool
Grainy	a flavor reminiscent of raw grain	28 mg/kg Butyraldehyde
Floral	The smell produced by a non-specific type of flower	1600 mg/kg phenethyl alcohol
Popcorn	aroma of popcorn	1300 mg/kg 2-Acetyl-1-pyrrolidine
Sweet	aroma of Demerara sugar	255 mg/kg 4-Hydroxy-2,5-dimethylfuran-3-one
Grassy	A green, slightly earthy, and slightly sweet flavor	26.3 mg/kg 1-Octanol
Woody	aroma of the dry, freshly cut wood smell	Toothpick
Oatmeal	aroma of cooked oatmeal	Quaker Oatmeal (Quaker, London, UK)
Dairy	aroma of uncooked milk	High-quality 2% Brand pasteurized milk
Corn	aroma of canned creamed corn	Cream Style Sweet Corn

**Table 3:** Comparison of the odor profiles and odor threshold values of the volatile compounds found in the three rice cultivars.

No.	Volatile Compounds	RI	Formula	CAS	Category	Odor Description ^1^	Odor Threshold ^2^ (μg/L)	Mean Content(μg/L) ^3^ ± Standard Deviation		
								A	B	C
1	Butanal	884	C_4_H_8_O	123-72-8	Aldehydes	Bready, Chocolate, Creamy, Pear, Vegetable, Fatty, Earthy, Woody, Pungent, Nutty, Wine-Like, Meaty, Spicy, Citrus, Herbaceous, Green, Apple, Floral, Musty, Cocoa, Fruity, Malty, Characteristic, Aldehyde Odor, Banana, Cocoa, Musty, Bready	9.0	1.87 ± 0.16	1.17 ± 0.48	2.20 ± 2.10
2	2-Butanone	898	C_4_H_8_O	78-93-3	Ketones	Ethereal, Ether, Fruity, Acetone, Camphor, Acetone-Like Odor, Sweet, Pleasant, Pungent, Moderately Sharp, Mint- Or Acetone-Like Odor, Pleasant, Camphoraceous	50,000.0	23.01 ± 2.21	17.87 ± 6.34	23.02 ± 12.50
3	2-Methylbutanal	907	C_5_H_10_O	96-17-3	Aldehydes	Musty, Coffee, Cocoa, Nutty, Almond, Fermented, Hazelnut, Malt, Malt, Burnt, Choking, Estery Apple, Fruity, Green Grass, Sour	1.0	0.45 ± 0.03	0.42 ± 0.12	0.31 ± 0.09
4	3-Methylbutanal	910	C_5_H_10_O	590-86-3	Aldehydes	Peach, Sour, Ethereal, Fatty, Aldehydic, Apple-Like Odor, Powerful Penetrating, Acrid Odor, Almond, Cocoa, Cheese, Chocolate, Malty	n.f.	7.57 ± 0.70	7.05 ± 3.64	6.08 ± 1.75
5	Pentanal	968	C_5_H_10_O	110-62-3	Aldehydes	Bready, Fermented, Berry, Malt, Fruity, Nutty, Acrid, Almond, Bitter, Oil, Pungent, Aldehyde, Banana, Grass, Lower Aldehyde, Oily, Green, Beany, Berry, Coffee, Green, Fatty	12.0	3.14 ± 0.87	1.66 ± 1.06	4.39 ± 4.48
6	Decane	1000	C_10_H_22_	124-18-5	Alkanes	Alkane	n.f.	0.35 ± 0.10	0.67 ± 0.09	0.74 ± 0.39
7	2-Butanol	1023	C_4_H_10_O	78-92-2	Alcohols	Oily, Sweet, Wine, Apricot, Has A Strong Vinous Odor, Sweet, Wine Odor Liquid, Strong, Pleasant Odor, Fruit, Medicine, Alcoholic	43,000.0	3.39 ± 0.91	2.84 ± 0.25	2.32 ± 1.87
8	Toluene	1029	C_7_H_8_	108-88-3	Aromatics	Paint, Sweet, Pungent, Benzene-Like Odor	2140.0	14.78 ± 2.55	14.12 ± 6.70	12.95 ± 4.60
9	1-Propanol	1035	C_3_H_8_O	71-23-8	Alcohols	Alcoholic, Fermented, Alcohol, Musty, Fusel, Pungent, Peanut, Similar To Ethanol, Alcohol, Candy	30,000.0	3.45 ± 2.14	3.97 ± 2.23	2.63 ± 0.63
10	2-Methyl-decane	1053	C_11_H_24_	6975-98-0	Alkanes	——	n.f.	1.13 ± 0.18	ND	1.23 ± 0.44
11	Undecane, 5-methyl-	1064	C_12_H_26_	1632-70-8	Alkanes	——	n.f.	1.04 ± 0.60	ND	1.87 ± 0.40
12	Hexanal	1072	C_6_H_12_O	66-25-1	Aldehydes	Leafy, Sweaty, Tallow, Fresh, Fatty, Fruity, Aldehydic, Green, Characteristic Fruity Odor (On Dilution), Strong, Green Grass Odor, Sharp, Aldehyde Odor, Apple, Fresh, Oil, Leaves, Vinous, Grassy	4.0	307.27 ± 33.46	ND	356.20 ± 210.81
13	Ethylbenzene	1111	C_8_H_10_	100-41-4	Aromatics	Aromatic, Pungent, Sweet, Gasoline-Like	140,000.0	1.95 ± 1.72	ND	3.28 ± 0.75
14	o-Xylene	1119	C_8_H_10_	95-47-6	Aromatics	Geranium, Sweet, Aromatic Odor	50.0	1.20 ± 0.19	1.66 ± 0.57	1.13 ± 0.94
15	2-n-Butyl furan	1122	C_8_H_12_O	4466-24-4	Heterocyclic	Mild, Wine, Sweet, Fruity, Spicy, Wet Hay, Noncharacteristic, Weak	n.f.	3.27 ± 0.45	1.66 ± 1.19	3.77 ± 2.58
16	1-Methoxy-2-propanol	1125	C_4_H_10_O_2_	107-98-2	Alcohols	Weak Pleasant Odor, Mild, Ethereal Odor	10,000.0	25.29 ± 6.71	29.70 ± 5.43	19.75 ± 16.89
17	Dodecane, 2,6,10-trimethyl-	1140	C_15_H_32_	3891-98-3	Alkanes	——	n.f.	0.78 ± 0.68	3.68 ± 1.73	1.82 ± 1.05
18	1-Butanol	1144	C_4_H_10_O	71-36-3	Alcohols	Oil, Vanilla, Fusel, Sweet, Balsam, Medicinal, Harsh Fusel Odor With Banana, Odor Similar To Amyl Alcohol, Rancid, Strong Characteristic, Mildly Alcoholic Odor, Wine, Floral, Fragrant, Fruity	500.0	8.84 ± 1.40	16.62 ± 17.70	12.43 ± 7.10
19	Undecane, 4-methyl-	1147	C_12_H_26_	2980-69-0	Alkanes	——	n.f.	0.89 ± 0.31	2.40 ± 1.12	1.07 ± 0.59
20	3-Hexanol	1157	C_6_H_14_O	623-37-0	Alcohols	Alcoholic, Ether, Medicinal, Solvent, Spice, Wine	820.0	2.27 ± 0.22	1.82 ± 1.10	1.52 ± 1.16
21	1-Penten-3-ol	1158	C_5_H_10_O	616-25-1	Alcohols	Butter, Pungent, Tropical, Horseradish, Green, Vegetable, Bitter, Fruity, Fish, Oxidized, Wet Earth, Burnt, Grass, Slightly Meaty, Mushroom	400.0	13.77 ± 3.29	6.53 ± 6.55	8.30 ± 5.88
22	2-Propenoic acid, butyl ester	1168	C_7_H_12_O_2_	141-32-2	Esters	Sharp, Fragrant, Biting Characteristic Odor, Fruity	n.f.	0.99 ± 0.28	0.44 ± 0.51	0.78 ± 0.54
23	2-Heptanone	1173	C_7_H_14_O	110-43-0	Ketones	Coconut, Soap, Herbal, Sweet, Woody, Fruity, Spicy, Cinnamon, Banana, Blue Cheese, Fruit, Green, Nut, Musty, Peardrops, Soapy, Butter	20.0	11.46 ± 2.37	6.79 ± 1.93	16.27 ± 14.37
24	Heptanal	1175	C_7_H_14_O	111-71-7	Aldehydes	Citrus, Ozone, Herbal, Fresh, Wine-Lee, Rancid, Fatty, Aldehydic, Green, Pungent Odor, Penetrating Fruity Odor, Citrus, Nut, Heavy, Oily, Planty Green, Putty, Milky	3.0	10.31 ± 1.32	6.63 ± 2.71	10.44 ± 5.17
25	1-Butanol, 3-methyl-	1205	C_5_H_12_O	123-51-3	Alcohols	Oil, Alcoholic, Burnt, Whiskey, Malt, Banana, Fusel, Fruity, Characteristic, Disagreeable, Burnt, Cocoa, Floral	250.0	7.14 ± 3.52	7.45 ± 1.99	6.52 ± 0.87
26	2-Pentylfuran	1222	C_9_H_14_O	3777-69-3	Heterocyclic	Butter, Green Bean, Vegetable, Earthy, Beany, Fruity, Metallic, Green, Greenbean, Butter, Floral, Pungent, Sweet, Buttery, Caramel, Fishy, Roasted, Popcorn	6.0	12.80 ± 1.44	8.50 ± 1.80	15.35 ± 9.76
27	1-Decanol	1237	C_10_H_22_O	112-30-1	Alcohols	Orange, Rose, Fat, Waxy, Fatty, Floral, Sweet, Clean, Watery, Fruity, Orange, Fat, Oil, Citrus, Soapy, Unripe Fruit	6.0	0.58 ± 0.19	1.16 ± 0.56	0.65 ± 0.33
28	Styrene	1243	C_8_H_8_	100-42-5	Alkenes	Balsamic, Gasoline, Floral, Sweet, Balsam, Plastic, Aromatic, Sweet, Floral	3.6	0.40 ± 0.39	1.19 ± 1.10	0.35 ± 0.45
29	1-Pentanol	1248	C_5_H_12_O	71-41-0	Alcohols	Oil, Balsamic, Vanilla, Fusel, Sweet, Fusel-Like, Fruit, Green, Pungent, Yeast, Fruit, Wax, Bread-Like	120.0	34.45 ± 4.85	28.07 ± 10.62	36.50 ± 17.34
30	1,2,3-Trimethylbenzene,	1266	C_9_H_12_	526-73-8	Aromatics	Distinctive, Aromatic Odor	n.f.	0.08 ± 0.13	0.17 ± 0.20	0.22 ± 0.16
31	2-Hexanol, 2,3-dimethyl-	1269	C_8_H_18_O	19550-03-9	Alcohols	——	n.f.	0.61 ± 0.33	0.21 ± 0.19	0.36 ± 0.45
32	3-Hydroxy-2-butanone	1271	C_4_H_8_O_2_	513-86-0	Ketones	Butter, Cream, Milky, Fatty, Creamy, Sweet, Dairy, Buttery, Bland, Woody, Yogurt Odor, Green Pepper, Fermented, Milky, Fatty	n.f.	15.09 ± 9.24	4.89 ± 1.75	9.38 ± 6.60
33	1-Decanol, 2-hexyl-	1272	C_16_H_34_O	2425-77-6	Alcohols	——	n.f.	0.50 ± 0.15	0.96 ± 0.37	0.54 ± 0.34
34	2-Octanone	1276	C_8_H_16_O	111-13-7	Ketones	Natural, Earthy, Gasoline, Weedy, Herbal, Woody, Bitter, Soap, Fatty, Green Cheese Aroma, Floral, Bitter, Fruity (Unripe Apple), Pleasant, Fat, Fragrant, Mold, Butter, Herb, Resin, Floral, Ketone, Musty, Soapy	41.0	17.01 ± 3.06	20.55 ± 3.75	22.08 ± 5.74
35	Octanal	1279	C_8_H_16_O	124-13-0	Aldehydes	Lemon, Citrus, Soap, Orange Peel, Fat, Waxy, Fatty, Aldehydic, Green, Strong, Fruity Odor, Fatty, Honey Odor On Dilution, Pungent Odor, Citrus-Like On Dilution, Aldehyde, Fruity, Pungent, Slightly Fragment, Soapy, Fresh, Citrus-Like	1.4	2.27 ± 0.43	1.33 ± 0.42	2.28 ± 1.72
36	1-Nonadecene	1306	C_19_H_38_	18435-45-5	Alkenes	——	n.f.	0.08 ± 0.04	0.18 ± 0.16	0.08 ± 0.16
37	(E)-2-Heptenal	1311	C_7_H_12_O	18829-55-5	Aldehydes	Soap, Vegetable, Fresh, Fatty, Pungent, Almond, Green, Fruity, Melting Plastic, Soapy, Tallow	13.0	2.77 ± 0.43	2.07 ± 0.81	2.97 ± 2.04
38	2,3-Octanedione	1316	C_8_H_14_O_2_	585-25-1	Ketones	Dill, Buttery, Broccoli, Cooked, Green, Roasted Nuts	n.f.	3.66 ± 0.78	2.92 ± 1.50	2.58 ± 0.72
39	5-Hepten-2-one, 6-methyl-	1328	C_8_H_14_O	110-93-0	Ketones	Apple, Mushroom, Citrus, Musty, Rubber, Nutty, Hazelnut, Bitter, Lemongrass, Powerful, Fatty, Green, Mushroom, Pepper, Strawberry, Sweet, Fruity, Lemongrass	50.0	1.54 ± 0.27	1.34 ± 0.37	1.16 ± 0.24
40	1-Hexanol	1350	C_6_H_14_O	111-27-3	Alcohols	Oil, Alcoholic, Ethereal, Resin, Fusel, Sweet, Fruity, Green, Characteristic, Sweet Alcohol, Pleasant, Banana, Herb, Fatty, Floral, Grassy	10.0	90.76 ± 14.00	120.41 ± 72.43	96.17 ± 19.66
41	2-Cyclopenten-1-one, 3-ethyl-2-hydroxy-	1377	C_7_H_10_O_2_	21835-01-8	Ketones	Butterscotch, Chocolate, Caramel, Smoky, Sweet, Maple, Fenugreek, Savory, Strong Caramel	n.f.	0.37 ± 0.25	0.36 ± 0.42	0.51 ± 0.38
42	2-Decanone	1381	C_10_H_20_O	693-54-9	Ketones	Floral, Orange, Peach, Fatty, Fruity, Musty	n.f.	1.08 ± 0.12	0.65 ± 0.25	0.99 ± 0.83
43	Nonanal	1385	C_9_H_18_O	124-19-6	Aldehydes	Citrus, Lime, Rose, Green, Fishy, Waxy, Fresh, Fatty, Peely, Aldehydic, Orris, Grapefruit, Orange-Rose Odor, Floral, Fat, Green, Lemon, Aldehyde, Floral, Grass, Slightly Pungent, Soapy, Tallow, Wax, Citrus-Like, Tallow, Fruity, Orange Peel	1.0	17.78 ± 4.03	13.83 ± 1.28	12.87 ± 2.67
44	3-Octanol	1392	C_8_H_18_O	589-98-0	Alcohols	Citrus, Nut, Moss, Herbal, Earthy, Woody, Melon, Minty, Mushroom, Spicy, Oil, Burnt, Chemical, Metal	18.0	0.68 ± 0.12	0.20 ± 0.40	0.33 ± 0.39
45	Formic acid, heptyl ester	1404	C_8_H_16_O_2_	112-23-2	Esters	Apple, Herbal, Waxy, Floral, Cucumber, Plum, Green	n.f.	0.09 ± 0.16	0.27 ± 0.20	0.10 ± 0.20
46	P-menthan-1-ol	1413	C_10_H_20_O	3901-95-9	Alcohols	——	n.f.	2.25 ± 0.75	0.80 ± 0.59	1.77 ± 1.85
47	Furfural	1416	C_5_H_4_O_2_	98-01-1	Aldehydes	Fragrant, Bread, Woody, Sweet, Baked, Almond, Peculiar Odor, Almond-Like Odor, Almond, Baked Potatoes, Burnt, Spice	280.0	0.42 ± 0.36	0.46 ± 0.32	0.20 ± 0.40
48	(E)-2-Octenal	1418	C_8_H_14_O	2548-87-0	Aldehydes	Nut, Fat, Herbal, Fresh, Green, Fatty, Banana, Waxy, Leaf, Cucumber, Green Leaf, Walnut, Almond, Fruity, Nutty	3.0	0.56 ± 0.07	0.40 ± 0.09	ND
49	1-Octen-3-one	1433	C_8_H_14_O	4312-99-6	Ketones	Herbal, Earthy, Metal, Musty, Mushroom, Dirty, Metallic	0.05	2.73 ± 0.45	3.88 ± 1.04	1.51 ± 1.48
50	Cis-linalool oxide	1436	C_10_H_18_O_2_	5989-33-3	Alcohols	Musty, Alcohol, Fenchyl, Camphor, Earthy, Floral, Green, Herbal, Wood	320.0	2.02 ± 0.23	1.42 ± 0.63	1.61 ± 0.36
51	1-Tetradecene	1442	C_14_H_28_	1120-36-1	Alkenes	Pleasant	n.f.	0.60 ± 0.06	0.74 ± 0.33	0.53 ± 0.05
52	1-Octen-3-ol	1447	C_8_H_16_O	3391-86-4	Alcohols	Raw, Fishy, Oily, Earthy, Fungal, Chicken, Mushroom, Green, Cucumber, Floral, Plastic, Soap, Fatty, Fruity, Grass, Perfumy, Sweet, Balsamic	1.0	43.75 ± 4.49	38.82 ± 6.43	47.65 ± 19.96
53	1-Heptanol	1452	C_7_H_16_O	111-70-6	Alcohols	Leafy, Coconut, Herbal, Peony, Strawberry, Chemical, Musty, Sweet, Woody, Violet, Green, Fragrant, Faint, Aromatic, Fatty, Mushroom	3.0	5.71 ± 0.85	4.12 ± 1.23	5.76 ± 2.93
54	2-Propylfuran	1481	C_7_H_10_O	4229-91-8	Heterocyclic	——	n.f.	0.57 ± 0.49	0.57 ± 0.45	0.46 ± 0.55
55	2-Ethyl-1-hexanol	1487	C_8_H_18_O	104-76-7	Alcohols	Citrus, Rose, Fresh, Floral, Oily, Sweet, Mild, Green, Rose, Citrus Fresh Floral Oily Sweet, Rosy	n.f.	11.54 ± 2.17	14.15 ± 5.82	13.33 ± 2.83
56	Decanal	1491	C_10_H_20_O	112-31-2	Aldehydes	Citrus, Soap, Orange Peel, Tallow, Waxy, Floral, Sweet, Aldehydic, Floral-Fatty Odor, Penetrating, Floral, Fried, Fatty, Green	9.0	0.74 ± 0.14	0.58 ± 0.21	0.47 ± 0.38
57	6-Undecanone	1521	C_11_H_22_O	927-49-1	Ketones	——	85.0	0.51 ± 0.10	0.09 ± 0.18	0.29 ± 0.37
58	4-Ethylcyclohexanol	1535	C_8_H_16_O	4534-74-1	Alcohols	——	n.f.	0.86 ± 0.16	0.80 ± 0.07	0.67 ± 0.16
59	2,2-Dimethyl-3-heptanone	1539	C_9_H_18_O	19078-97-8	Ketones	——	n.f.	1.90 ± 0.34	1.05 ± 0.73	1.85 ± 1.37
60	1-Octanol	1555	C_8_H_18_O	111-87-5	Alcohols	Burnt, Orange, Rose, Waxy, Chemical, Metal, Aldehydic, Mushroom, Green, Fresh, Aromatic, Bitter Almond, Burnt Matches, Fat, Floral, Moss, Nut, Green Herbaceous, Citrus-Like	42.0	3.68 ± 0.17	2.70 ± 0.76	3.47 ± 1.74
61	Isophorone	1580	C_9_H_14_O	78-59-1	Ketones	Cooling, Tobacco, Cedarwood, Leather, Musty, Woody, Sweet, Fruity, Camphoraceous, Camphor, Green, Peppermint-Like Odor, Spice	2000.0	1.26 ± 1.12	1.74 ± 1.29	0.96 ± 1.12
62	Myristic acid vinyl ester	1594	C_16_H_30_O_2_	5809-91-6	Esters	——	n.f.	0.58 ± 0.06	0.52 ± 0.38	0.23 ± 0.29
63	Heptacosane	1622	C_27_H_56_	593-49-7	Alkanes	——	n.f.	ND	ND	0.22 ± 0.39
64	(E)-2-Decenal	1635	C_10_H_18_O	3913-81-3	Aldehydes	Orange, Coriander, Rose, Tallow, Waxy, Oily, Fatty, Earthy, Floral, Aldehydic, Mushroom, Green	n.f.	0.81 ± 0.09	0.43 ± 0.51	0.64 ± 0.45
65	Furan, 2-hexyl-	1755	C_10_H_16_O	3777-70-6	Heterocyclic	——	n.f.	0.24 ± 0.01	0.16 ± 0.04	0.11 ± 0.13
66	3-Tridecanone	1770	C_13_H_26_O	1534-26-5	Ketones	——	n.f.	0.27 ± 0.24	0.26 ± 0.17	0.31 ± 0.02
67	Phenol, 4-(1-methylpropyl)-	1832	C_10_H_14_O	99-71-8	Aromatics	——	n.f.	0.13 ± 0.06	0.03 ± 0.06	0.09 ± 0.05
68	Propanoic acid, 2-methyl-, 3-hydroxy-2,2,4-trimethylpentyl ester	1862	C_12_H_24_O_3_	77-68-9	Esters	——	n.f.	0.29 ± 0.25	0.85 ± 0.32	0.44 ± 0.06
69	2,2,4-Trimethyl-1,3-pentanediol diisobutyrate	1873	C_16_H_30_O_4_	6846-50-0	Esters	——	n.f.	3.29 ± 0.99	3.34 ± 1.52	1.97 ± 0.54
70	2-Acetyl-1h-pyrrole	1956	C_6_H_7_NO	1072-83-9	Heterocyclic	Licorice, Coumarin, Bread, Musty, Nutty, Walnut, Bread, Cocoa, Hazelnut, Caramel, Fatty, Mothball, Oily, Putty	170,000.0	0.06 ± 0.06	ND	0.02 ± 0.00
71	2(3H)-Furanone, dihydro-5-pentyl-	2013	C_9_H_16_O_2_	104-61-0	Esters	Peach, Coconut, Waxy, Oily, Creamy, Sweet, Buttery	7.0	0.61 ± 0.16	0.65 ± 0.15	0.71 ± 0.24
72	2-Pyrrolidinone	2020	C_4_H_7_NO	616-45-5	Ketones	——	n.f.	0.07 ± 0.13	0.06 ± 0.12	0.29 ± 0.11
73	2-Tetradecanone	2122	C_14_H_28_O	2345-27-9	Ketones	——	n.f.	0.02 ± 0.03	0.07 ± 0.03	0.06 ± 0.02
74	Benzofuran, 2,3-dihydro-	2361	C_8_H_8_O	496-16-2	Heterocyclic	——	n.f.	0.18 ± 0.02	0.17 ± 0.12	0.12 ± 0.08

^1^ Odor description found in the literature with database (Flavornet; FlavorDB2; The LRl and Odor Database). ^2^ All odor thresholds were obtained from: Odor & Flavor Detection Thresholds in Water (In Parts per Billion, lg/L) [11,15,26,35,40,41,42,43,44,45,46,47,48,49,50]. ^3^ The values are based on a semi-quantitative analysis using the area of 2-methyl-3-heptanone (IS). RI, retention index, which was calculated referred to the retention time of c5-c28 n-alkanes under the same conditions. “——”, no odor description information was found in the literature. “n.f.”, data was not found in the literature. “ND”, not detected.

**Table 4:** Analysis of variance of differential volatile metabolites.

Volatile Compounds	RI	ANOVA *p*-Value	Q-Value	Mean Content(μg/kg) * ± Standard Deviation		
				A	B	C
2-Methyldecane	1053	0.012	0.363	1.13 ± 0.18	2.92 ± 1.26	1.23 ± 0.44
Dodecane, 2,6,10-trimethyl-	1140	0.035	0.623	0.78 ± 0.68	3.68 ± 1.73	1.82 ± 1.05
P-menthan-1-ol	1413	0.018	0.498	2.25 ± 0.75	0.80 ± 0.59	1.77 ± 1.85
(E)-2-Octenal	1418	0.001	0.333	0.56 ± 0.07	0.40 ± 0.09	ND
1-Octen-3-one	1433	0.044	0.623	2.73 ± 0.45	3.88 ± 1.04	1.51 ± 1.48
6-Undecanone	1521	0.022	0.498	0.51 ± 0.10	0.09 ± 0.18	0.29 ± 0.37
Heptacosane	1622	0.025	0.517	ND	ND	0.22 ± 0.39
2-Acetyl-1h-pyrrole	1956	0.007	0.498	0.06 ± 0.06	ND	0.02 ± 0.00

* The values are based on a semi-quantitative analysis using the area of 2-methyl-3-heptanone (IS). “ND”, not detected.

**Table 5:** GC-IMS analysis of volatile components of the three aromatic rice samples.

No.	Volatile Compounds	RI ^1^	RT/s ^2^	Drift Time/ms ^3^	Peak Volume		
					A	B	C
1	(+)-Limonene	1189.3	433.309	1.65766	47.32	21.00	18.45
2	1,2-Ethanediol	1611.1	1224.033	1.15889	193.75	156.70	307.64
3	1-Butanol-D	1150.3	376.987	1.3813	1348.39	1055.02	1285.03
4	1-Butanol-M	1150.8	377.739	1.18107	120.26	102.56	132.41
5	1-Butanol, 3-methyl-, acetate	1098.9	313.929	1.29261	94.11	55.98	48.65
6	1-Hexanal-D	1092.5	307.402	1.5605	2004.85	1105.25	812.02
7	1-Hexanal-M	1090.7	305.662	1.2717	410.76	168.15	199.22
8	1-Hexanol-D	1370.6	730.557	1.32995	2577.51	1732.20	1479.16
9	1-Hexanol-M	1368.9	727.847	1.6473	858.88	403.07	317.09
10	1-Octen-3-ol	1476.4	916.663	1.1603	140.36	152.57	455.21
11	1-Pentanol-D	1257.5	546.758	1.51302	3641.90	2330.38	2190.92
12	1-Pentanol-M	1258.7	548.929	1.25232	566.51	535.20	532.15
13	1-Propanol-D	1043.8	263.684	1.2563	1635.42	2108.75	2257.31
14	1-Propanol-M	1044.8	264.48	1.10846	77.39	82.29	62.42
15	2,3-Bbutanedione	927.4	190.575	1.17797	2020.23	2355.91	2300.38
16	2,6-Dimethyl pyrazine-D	1359.2	712.777	1.53432	96.20	87.06	325.36
17	2,6-Dimethyl pyrazine-M	1359.4	713.102	1.14292	42.39	39.75	82.61
18	2-Acetylpyridine	1569.9	1120.373	1.11215	138.31	152.23	372.34
19	2-Butanone	912	183.29	1.24516	1833.67	1415.23	1477.38
20	2-Heptanone-D	1192	437.464	1.62967	979.16	439.76	427.46
21	2-Heptanone-M	1188.7	432.325	1.26116	853.13	439.76	521.98
22	2-Hexanone-D	1097	311.827	1.50233	537.91	431.80	459.12
23	2-Hexanone-M	1095	309.868	1.19273	449.86	393.95	430.29
24	(E)-2-Hhexen-1-ol	1348.6	696.854	1.17499	186.52	159.96	159.59
25	2-Methylbutanoic acid, methyl ester	1028.1	250.954	1.18974	241.93	282.58	220.97
26	2-Methylpyrazine	1292.7	616.299	1.06994	894.63	288.30	321.60
27	2-Nonanone-D	1398	774.751	1.88186	49.58	47.39	49.36
28	2-Nonanone-M	1401.7	780.967	1.87851	43.77	42.12	39.74
29	2-Octanone-D	1295	621.087	1.75701	368.72	282.99	311.93
30	2-Octanone-M	1292.4	615.705	1.33009	88.85	88.59	90.09
31	2-Pentanone	996	226.78	1.3725	109.65	56.16	64.99
32	2-Propanol	920.1	187.091	1.22384	1142.23	1759.81	1455.53
33	3-Methyl butyl acetate	1149.9	376.536	1.31347	53.16	29.85	34.89
34	Acetic acid	1477.7	919.242	1.0518	2331.29	2504.44	4153.42
35	Acetic acid ethyl ester-D	914.3	184.354	1.32401	348.72	316.74	274.78
36	Acetic acid ethyl ester-M	918.1	186.15	1.09431	56.30	23.46	35.04
37	Acetophenone-D	1688.5	1445.218	1.17229	988.12	1180.18	4038.73
38	Acetophenone-M	1688	1443.765	1.38686	44.25	54.32	115.64
39	α-Pinene	992.3	224.692	1.29579	221.34	157.79	169.56
40	Benzaldehyde	1565.4	1109.612	1.14581	200.18	124.63	161.89
41	Butanal	894.9	175.517	1.28753	44.73	35.52	39.40
42	Butanoic acid, ethyl ester	1018.6	243.498	1.56902	171.15	179.31	126.95
43	Cyclohexanone	1292.1	614.912	1.15711	184.07	77.66	83.86
44	Ethanol	939.2	196.365	1.13225	3471.54	3298.02	3895.87
45	Ethyl acrylate	1025.7	249.08	1.41748	36.99	37.05	39.45
46	Ethyl butanoate	991	223.924	1.19944	1327.66	714.71	558.32
47	Ethyl hexanoate-D	1241.7	517.989	1.33922	361.29	366.11	920.86
48	Ethyl hexanoate-M	1242.7	519.833	1.78996	187.21	163.97	313.61
49	Ethyl octanoate	1401.9	781.337	1.47904	452.88	429.68	319.57
50	Ethyl pentanoate	1172.2	407.663	1.26934	121.34	88.84	99.17
51	Heptanal	1131.5	352.605	1.31628	30.45	24.15	26.67
52	n-Pentanal	990.2	223.486	1.41859	370.03	94.34	55.40
53	Propanal-D	791.5	135.056	1.06433	422.97	489.08	569.58
54	Propanal-M	790.4	134.684	1.14053	601.24	489.08	435.88
55	Styrene	1296.4	623.002	1.41216	326.33	208.17	181.32

M, monomers; D, dimers. ^1^ The retention index (RI) calculated using n-ketones C4–C9 as external standard on FS-SE-54-CB column. ^2^ The retention time (RT) of identified volatiles in the capillary GC column. ^3^ The drift time in the drift tube.

## Data Availability

The original contributions presented in this study are included in the article. Further inquiries can be directed to the corresponding author.

## Abbreviations

The following abbreviations are used in this manuscript:

VOCs	Volatile organic compounds
HS-SPME-GC-MS	Headspace solid-phase microextraction gas chromatography mass spectrometry
HS-GC-IMS	Headspace gas chromatography-ion mobility spectrometry
GC-O	Gas chromatography-olfactometry
RI	Retention index
ROAV	Relative odor activity value
PCA	Principal component analysis
ANOVA	Analysis of variance
OPLS-DA	Orthogonal Partial Least Squares-Discriminant Analysis
VIP	Variable Importance in the Projection
RT	Retention time
QDA	Quantitative descriptive analysis
LAV	Laboratory Analysis View software
